# Complete genome sequences of *Cytobacillus* sp., *Domibacillus* sp., *Enterobacter* sp., *Neisseria* sp., *Pseudomonas* sp., and *Streptococcus* sp. strains from human clinical infections collected at diagnostic units in 2020

**DOI:** 10.1128/mra.01040-23

**Published:** 2024-05-29

**Authors:** Judit Szarvas, Sidsel Nag, Saria Otani, Laura Elmlund Kohl Birkedahl, Frederik Duus Møller, Solomon Asante-Sefa, Denise Daley, Natalie Weiler Gustafson, Mie Møller, Anthony Onipede, Silva Tafaj, Frank M. Aarestrup

**Affiliations:** 1 Research Group for Genomic Epidemiology, National Food Institute, Technical University of Denmark, Lyngby, Denmark; 2 Sekondi Public Health Laboratory, Effia Nkwanta Regional Hospital, Ghana Health Service, Sekondi-Takoradi, Ghana; 3 Department of Microbiology, PatWest Laboratory Medicine, Fiona Stanley Hospital, Murdoch, Australia; 4 Departamento de Bacteriologia, Laboratorio Central de Salud Publico, Avenida Venezuela y Tte Escurra, Asunción, Paraguay; 5 Dronning Ingrids Hospital, Nuuk, Greenland; 6 Obafemi Awolowo University, Ile-Ife, Nigeria; 7 Microbiology Department, University Hospital “Shefqet Ndroqi”, Tirana, Albania; University of Maryland School of Medicine, Baltimore, Maryland, USA

**Keywords:** pathogens

## Abstract

Members of *Bacillota* and *Pseudomonadota* phyla are frequently considered bacterial infectious agents in humans. As part of a large sequencing project of clinically relevant pathogens, we hybrid-assembled complete genomes of *Cytobacillus*, *Domibacillus*, *Enterobacter*, *Neisseria*, *Pseudomonas,* and *Streptococcus* species isolated from clinical specimens.

## ANNOUNCEMENT

Antimicrobial resistance (AMR) in clinically relevant bacteria is a worldwide concern, causing heightened economic and disease burden (1, 2). We conducted the research project “Two Weeks in the World” (TWIW), to assess the species and AMR-conferring gene diversity in clinical pathogenic isolates across the world in 2020 (3). Besides releasing draft genomes for all sequenced isolates, we performed hybrid assemblies for isolate genomes that lacked a closely related reference genome. Here, we report the complete genomes of four *Bacillota* and five *Pseudomonadota* (Table 1), collected in Albania (*n* = 1), Australia (*n* = 1), Ghana (*n* = 1), Greenland (*n* = 2), Nigeria (*n* = 2), and Paraguay (*n* = 1).

**TABLE 1:** Properties of the complete genomes

Isolate no.	*1000418*	*1000833*	*1000888*	*1001019*	*1001157*	*1001937*	*1001991*	*1002640*
Type	*Cytobacillus* sp.	*Neisseria* sp.	*Streptococcus sanguinis*	*Streptococcus* sp.	*Domibacillus* sp.	*Pseudomonas* sp.	*Neisseria* sp.	*Enterobacter hormaechei*
Bio sample metadata								
Country of isolation	Ghana	Greenland	Greenland	Australia	Albania	Nigeria	Nigeria	Paraguay
Strain supplier	Sekondi Public Health Laboratory	Dronning Ingrids Hospital	Dronning Ingrids Hospital	Fiona Stanley Hospital	University Hospital Shefqet Ndroqi	Obafemi Awolowo University Teaching Hospital	Obafemi Awolowo University Teaching Hospital	Hospital La Costa
City	Sekondi	Nuuk	Nuuk	Murdoch	Tirana	Ile-Ife	Ile-Ife	Asunción
Source	Blood	Expectorate	Expectorate	Urine	Swab (wound)	Blood	Sputum	Swab (wound)
Date of sampling from a patient	2020-03-16	2020-05-16	2020-05-25	2020-06-21	2020-03-06	2020-10-29	2020-11-09	2020-11-18
Cultivation media	Blood agar	Chocolate agar	Blood agar	Blood agar	Blood agar	Blood agar	Blood agar	Blood agar
Illumina sequencing								
Raw-reads accession	ERR10438045	ERR10438049	ERR10438146	ERR10431340	ERR10430225	ERR10438816	ERR10438842	ERR10431730
Total number of trimmed reads	4,709,974	993,090	742,924	1,582,510	1,286,740	2,128,422	2,047,614	12,094,220
Genome coverage	148.6×	51.2×	39.0×	98.4×	32.6×	52.6×	117.4×	344.0×
Nanopore sequencing								
Raw-reads accession	ERR12041378	ERR12041383	ERR12075676	ERR12075678	ERR12075675	ERR12041388	ERR12041384	ERR12075677
Total number of trimmed reads	1,068,084	224,055	150,636	503,851	334,954	79,872	144,312	677,901
N50 (bp)	18,160	33,985	24,383	9,194	12,113	33,134	29,769	30,811
Genome coverage	972.8×	1,125.6×	1,123.3×	1,673.4×	637.9×	364.7×	863.2×	1,625.8×
Assembly								
Assembler	Unicycler	Unicycler	Unicycler	Flye	Flye	Unicycler	Unicycler	Unicycler
Assembly accession	GCA_032341175.1	GCA_032388755.1	GCA_032387735.1	GCA_032340315.1	GCA_032341995.1	GCA_032463525.1	GCA_032342915.1	GCA_032389435.1
Assembly total length (bp)	4,468,467	2,579,073	2,587,876	2,185,353	5,309,781	5,662,787	2,401,490	4,895,951
Number of contigs	1	1	2	2	2	2	1	1
Chromosome length (bp)	4,468,467	2,579,073	2,520,163	2,161,951	5,309,781	5,564,925	2,401,490	4,895,951
G + C content (%)	38.5	51.2	43	39.5	43.5	64	49.5	55
Number of genes	4,405	2,506	2,576	2,065	5,119	4,975	2,291	4,672
Antimicrobial resistance genes	-	-	msrD, mefA	msrD, mefA, tetW	-	-	aph(3″)-Ib, aph (6)-Id, tetB, penA	qxA, oqxB, blaACT-7

Clinical isolates were collected and stored by the project’s Partner Units according to the standard clinical microbiology operation procedures. This study was exempt from ethical approval based on the assessment of the Committees of the Capital Region of Denmark (received on 2020-04-06). Our partners followed their respective national guidelines and regulations during their participation in the study.

Isolates were transferred on coal swabs, and cultured on blood agar or chocolate agar (1000833), one sample (1001157) with CO_2_. Subcultured until clean, with experienced laboratory professionals choosing colonies based on expected species provided by the strain supplier (3). DNA for both short- and long-read sequencing was extracted from overnight cultures from the same sub-culture.

For short-read sequencing, DNA was extracted using a Qiagen DNeasy Blood & Tissue kit (Qiagen, Netherlands) according to the manufacturer’s protocol. DNA concentrations were quantified using Invitrogen’s Qubit dsDNA high-sensitivity assay kit (Carlsbad, USA). Libraries were prepared according to the Illumina NexteraXT DNA Library Prep (Illumina Inc., CA, USA) using standard normalization. All samples were sequenced on an Illumina NextSeq 500 platform, with paired-end sequencing (300 cycles) on medium output flowcells. Quality control and trimming of the short-read raw data were performed with FoodQCPipeline v1.5 (https://bitbucket.org/genomicepidemiology/foodqcpipeline) using the default settings.

For long-read sequencing, high molecular weight DNA was extracted using Quick-DNA HMW MagBead Kit (Zymo Research, D6060). The DNA was sequenced on Oxford Nanopore platform GridION using ligation library protocol with 12 chemistry on flowcell R9.4 (SQK-NBD112.24) following the manufacturer’s instructions without fragmentation or size selection steps (Oxford Nanopore Technology, UK). The reads were base-called using Guppy base-caller v5.0.7 and the “super high accuracy” function. Quality was assessed with NanoPlot v1.33.0 (4) and trimmed to a minimum read length of 1,000 bp by Filtlong v0.2.0 (https://github.com/rrwick/Filtlong).

Genomes were assembled using Unicycler v0.5.0 (5) or Flye v2.9 (6) with polishing with medaka v1.7.2 (https://github.com/nanoporetech/medaka) and Polypolish v0.5.0 (7) once each. The circularization of the contigs was determined by the assembler software and confirmed by visualization with Bandage v0.9.0 (8). The contigs were not rotated. Taxonomy was assigned based on the results from the de_novo_wf of GTDB-Tk v2.1.0 (9). Genome completeness was estimated by busco v5.4.5 (10) and the genomes were annotated by PGAP v6.6 (11). AMR gene prediction was done using ResFinder v4.1 with the “-s other” flag on database dated 2023-03-11 (https://bitbucket.org/genomicepidemiology/resfinder_db) (12). Default parameters were used except where otherwise noted.

## Data Availability

The complete genomes reported in this announcement have been deposited in DDBJ/ENA/GenBank under BioProject no. PRJEB56918. The versions described in this paper are the first versions. The accession numbers for both raw data and assemblies are presented in Table 1, hyperlinked to the publicly available records.

## References

[1] Murray CJL, Ikuta KS, Sharara F, Swetschinski L, Robles Aguilar G, Gray A, Han C, Bisignano C, Rao P, Wool E, et al.. 2022. Global burden of bacterial antimicrobial resistance in 2019: a systematic analysis. Lancet 399:629–655. doi:10.1016/S0140-6736(21)02724-0 35065702 PMC8841637

[2] WHO. 2022. Global antimicrobial resistance and use surveillance system (GLASS) report 2022. Geneva

[3] Nag S, Larsen G, Szarvas J, Birkedahl LEK, Gulyás GM, Ciok WJ, Lagermann TM, Tafaj S, Bradbury S, Collignon P, et al.. 2023. Whole genomes from bacteria collected at diagnostic units around the world 2020. Sci Data 10:628. doi:10.1038/s41597-023-02502-7 37717051 PMC10505216

[4] De Coster W, Rademakers R. 2023. NanoPack2: population-scale evaluation of long-read sequencing data. Bioinformatics 39:btad311. doi:10.1093/bioinformatics/btad311 37171891 PMC10196664

[5] Wick RR, Judd LM, Gorrie CL, Holt KE. 2017. Unicycler: resolving bacterial genome assemblies from short and long sequencing reads. PLoS Comput Biol 13:e1005595. doi:10.1371/journal.pcbi.1005595 28594827 PMC5481147

[6] Kolmogorov M, Yuan J, Lin Y, Pevzner PA. 2019. Assembly of long, error-prone reads using repeat graphs. Nat Biotechnol 37:540–546. doi:10.1038/s41587-019-0072-8 30936562

[7] Wick RR, Holt KE. 2022. Polypolish: short-read polishing of long-read bacterial genome assemblies. PLOS Comput Biol 18:e1009802. doi:10.1371/journal.pcbi.1009802 35073327 PMC8812927

[8] Wick RR, Schultz MB, Zobel J, Holt KE. 2015. Bandage: interactive visualization of de novo genome assemblies. Bioinformatics 31:3350–3352. doi:10.1093/bioinformatics/btv383 26099265 PMC4595904

[9] Chaumeil P-A, Mussig AJ, Hugenholtz P, Parks DH. 2022. GTDB-Tk v2: memory friendly classification with the genome taxonomy database. Bioinformatics 38:5315–5316. doi:10.1093/bioinformatics/btac672 36218463 PMC9710552

[10] Seppey M, Manni M, Zdobnov EM. 2019. BUSCO: assessing genome assembly and annotation completeness. Methods Mol Biol 1962:227–245. doi:10.1007/978-1-4939-9173-0_14 31020564

[11] Li W, O’Neill KR, Haft DH, DiCuccio M, Chetvernin V, Badretdin A, Coulouris G, Chitsaz F, Derbyshire MK, Durkin AS, Gonzales NR, Gwadz M, Lanczycki CJ, Song JS, Thanki N, Wang J, Yamashita RA, Yang M, Zheng C, Marchler-Bauer A, Thibaud-Nissen F. 2021. RefSeq: expanding the prokaryotic genome annotation pipeline reach with protein family model curation. Nucleic Acids Res 49:D1020–D1028. doi:10.1093/nar/gkaa1105 33270901 PMC7779008

[12] Bortolaia V, Kaas RS, Ruppe E, Roberts MC, Schwarz S, Cattoir V, Philippon A, Allesoe RL, Rebelo AR, Florensa AF, et al.. 2020. ResFinder 4.0 for predictions of phenotypes from genotypes. J Antimicrob Chemother 75:3491–3500. doi:10.1093/jac/dkaa345 32780112 PMC7662176

